# Gossypiboma: Retained Surgical Sponge after a Gynecologic Procedure

**DOI:** 10.1155/2010/917626

**Published:** 2010-08-03

**Authors:** Aziz Sümer, M. Ali Çarparlar, Ömer Uslukaya, Vedat Bayrak, Çetin Kotan, Ozgur Kemik, Umit Iliklerden

**Affiliations:** Department of Surgery, Faculty of Medicine, Yüzüncü Yil University, 65200 Van, Turkey

## Abstract

We report on a case of gossypiboma. A 54-year-old woman was admitted to our hospital with abdominal mass. She had undergone a caesarean operation 23 years previously. The mass in the right abdominal quadrant was suspected by abdominal computed tomography and magnetic resonance imaging. The mass was removed by laparotomy excision and the final diagnosis was gossypiboma.

## 1. Introduction

Gossypiboma is derived from gossypium (“cotton” in Latin) and boma (“place of concealment” in Swahili) and is a rare complication of surgery [1–4]. The retention of surgical sponges in body cavities is a preventable condition that has not been very frequently reported due to possible medicolegal concerns [2]. Reports in the literature are generally related to intraabdominal foreign object, but a few reports show the gossypiboma localizing other parts of the body like intrathoracic and pericardial spaces [5, 6]. A foreign body can trigger a granulomatous reaction, so the retained surgical gauze was indicated by foreign-body reaction. Because of the fact that gossypibomas appear years after surgery and have nonspecific symptoms, they are usually identified on imaging sequences [4, 7]. Delays in diagnosis and treatment may cause serious morbidity and may lead to mortality [2, 3, 8]. 

 We herein report a gossypiboma resulting from 3 retained surgical swabs that had been left in peritoneum for 23 years after a caesarean operation.

## 2. Case Report

**Figure 1:**
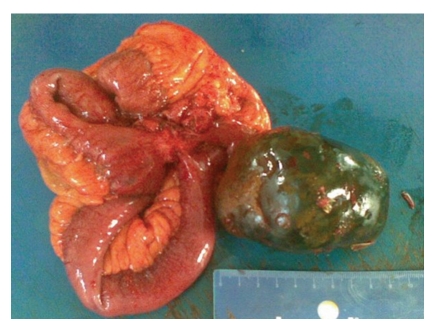
Operative view of the conglomerated mass after removal.

**Figure 2:**
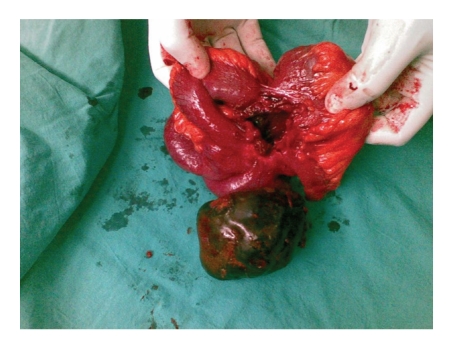
The view of the mass extending into ileum.

A 54-years-old female patient was admitted to our policlinic with intermitent abdominal pain and complaint of rigidity around her umbilicus. It was learned from her past history that she had a Caesarean operation 23 years ago. On her a physical examination a pfannenstiel incision scar and a 15 by 10 cm diameter rigid, mobile mass located right abdominal cavity were found. On abdominal computed tomography (CT) scan the 6 cm-by-6 cm in diameter calcific mass had smooth edges and a central lipophilic density located at right quadrant was thought as a lipoma. On magnetic resonance imaging (MR) scan the 43-by-42 mm diameter mass that did not take intravenous contrast agent was evaluated as a gallbladder stone or myoma uteri. We operated on the patient. The abdominal cavity was explored. On exploration in the right abdominal quadrant the 15-by-10 by 6 cm diameter a rigid and mobile mass that conglomerated with ileum, transvers colon and caecum was found (Figure 1). No dilated bowel ansa found at the superior site of the mass. A fistula formation was observed among caecum, colon and ileum. It was observed that mass extend into ileum (Figure 2). On examination after removal of the mass 3 gauze were found inside it. Segmentary resection and primary anastomosis were made for ileal damage. Colon and caecum were repaired with primary suture after debridement. Patients were discharged without any complications.

## 3. Discussion

Gauze, ped, compress, and clamps are the most forgotten materials after surgical operations. The incidence of gossypiboma is difficult to estimate because of not being reported, but it has been reported that varies from 0.01% to 0.001%. Most of the gossypiboma are identified only after abdominal or pelvic surgery [6, 7]. Retained sponges are most frequently observed in patients with obesity, during emergency operations and following laparoscopic interventions. Gossypiboma is most frequently diagnosed in the intraabdominal cavity; however, it can also be seen in paraspinal muscles, intrathoracic region, legs, shoulders and pericardial space [2, 6]. 

 Gossypiboma induce two types of reaction. First is exudative in nature and leads to formation of abscess. An aseptic fibrinous response, which creates adhesions and encapsulation and eventually results in the developmentof a foreign-body granuloma [7]. 

Abdominal gossypibomas may be asymptomatic or may present with abdominal pain, abdominal mass, intestinal obstruction, gastrointestinal hemorrhage, intraabdominal sepsis, granulomatous peritonitis, and fistulization into surrounding structures [9].

Because the symptoms of gossypiboma are usually nonspecific and may appear years after surgery, the diagnosis of gossypiboma usually comes from imaging studies and a high index of suspicion. In most countries, surgical sponges contain radiopaque material that facilitates detection by standard abdominal radiography. Such sponges can also be identified readily in CT images. However, surgical sponges without radiopaque markers are still used in many institutions, and this type of sponge is very difficult to identify by using standard radiographic and CT imaging. Therefore, retained surgical foreign bodies often make diagnosis intractable [7].

The granulomatous reaction and surrounding tissues caused by foreign body may reach huge volume and, may lead to misdiagnosis as a tumor. This huge mass sometimes causes to bowel occlusion. If the gossypiboma consist of metal, possiblity of perforation will increase and, mortality due to this condition increases [10]. 

After the diagnosis of gossypiboma is confirmed, removal of the retained sponge surgically, endoscopically or laparoscopically is accomplished in order to prevent severe morbidity or mortality may lead to death [1].

In conclusion, gossypiboma can be diagnosed early postoperative stage providing that usig materials having radiopaque markers. However, materials having radiopaque markers are not used in the most of health service in our country. In order to prevent these types of complications, we have to control all of the surgical materials before and after surgery, which is the main principle in all procedures.
